# A Remarkable Obstetrical Case

**Published:** 1859-04

**Authors:** 


### *A Remarkable Obstetrical Case.*

JANUARY 19th, 1859.

PROF. WHITE:

My Dear Sir,—Pardon me for the liberty I have taken in addressing you at this time; and although my subject is rather a peculiar one, yet I hope it may not prove altogether uninteresting to you. In our quiet village, we have a fellow who is pretending to practice medicine under the name of an "Eclectic Physician;" does a very small business, and is quite an active member of the Methodist church. Before relating the ludicrous case connected with his professional career, I will state that it is only one among a host of others, about as ridiculous as this, and goes to show plainly that "empiricism" is tolerated in our midst, and that people are anxious to be humbugged, and are not content unless they are.

Some two weeks since, this "eclectic physician" was sent for to attend Mrs. W., living three miles from this village, who was about being confined; he, of course, started for the scene of action with all possible speed. Arriving in due time, he at once proceeded to examine the case. After making a very close and precise investigation, he decided that there was something wrong; and as he expressed it, *the womb was trying to come into the world.* He informed the lady in labor, and those present, that it was his duty to sit by the bed-side, and when a pain came on, he must push against the *womb and keep it from coming into the world;* he accordingly put his theory into practice, and for ten or twelve long hours strongly resisted the natural and kind efforts of nature. Finally, finding himself a good deal exhausted, and his patient becoming uneasy, as well as the other ladies present, it was decided that another physician should be called in, and particular instructions was given to the messenger to tell the other doctor to bring his instruments, for there had got to be *something cut away.* Accordingly the doctor started with his entire outfit for a serious case. Upon arriving at the house, the "eclectic physician" called him aside, and informed him that he had a desperate case on hand, for, said he, *the womb is trying to come down into the world, and I cannot stop it, and I do not think the child can ever be born, for the mouth of the womb is so small that I can only get my little finger into it, and the pains do not dilate it a particle.* As the lady and the friends were becoming very anxious that something should be done, if possible, the doctor last called, (who, by the way, was a regular physician of good standing,) proceeded to examine the case, and to his utter surprise, found a perfectly natural case of labor, with a breech presentation, which the "eclectic physician" had taken for the womb, and had absolutely inserted his little finger into the *child's rectum, and called it the mouth of the uterus.* The doctor called last informed the lady that there was no difficulty about the case, and in a few moments she was delivered of a child, but of course dead, being bruised and horribly marked by the efforts used to keep the *womb from coming into the world.*

Now this is all true to a letter, not in the least exaggerated. Dr. C. B. Hutchins of your own city is familiar with all the parties, but I will call no names. I would like if Prof. Flint could see this, and as many others of your medical friends as you see proper, and you are at liberty to do with it as you please. * * * *

Prof. White showed us some time ago the above amusing letter, which we have thought not inappropriate for the pages of our Journal. In spite of the sad mistake in diagnosis, we cannot but admire the indomitable perseverence and energy of the valant eclectic who stormed the *breech* so manfully for between ten or twelve hours. Truly it must have been a terrible conflict! man against woman! Interesting, however, in a physiological point of view, as showing the superiority of the involuntary over the voluntary muscle, as the uterus finally conquered the opposing force, pushing the body of the devoted infant into the world, the soul of which, the ruthless practitioner had succeeded in pushing out.

It makes us ashamed of our race, because they will suffer it, and of ourselves, because we cannot prevent it; but there is no doubt but that the human family will be quacked and humbugged forever. They know so little of medical science; worse than nothing indeed, for most persons imagine that they are learned in some disease, and civilization is so far advanced, that the world at large will neither confess nor even believe in their ignorance. Would to heaven that this were confined to the least cultivated classes of society! Then we might hope that those above them would make laws which would effectually restrain the most glaring and rampant phases of charlatanism. But this is not so; multitudes upon multitudes of our most educated people are the constant patrons of medical humbug. A quack, by whose presence our city is now honored, and who does not conceal or apparently wish to conceal his humbuggery, is patronized largely, and, we blush to write, receives an occasional visit from persons in "high places." But such is the world, "*vive la bagatelle.*" We can never compete with irregulars; can never drive all mankind to the true faith; and we should have known it, if we did not, when we embarked upon the troubled sea of a professional life.

---

*Compliment to Dr. Lemon.*—The following correspondence should have appeared in our last issue, but owing to a mistake on the part of the committee, it was not presented to the proper person, in time. We take great pleasure in now publishing this well merited compliment:

BUFFALO, Feb. 18th, 1859.

DR. BENJ. H. LEMON:

Dear Sir—The students of the medical department of the University of Buffalo, as a token of their esteem and their appreciation of the efficient discharge of your duties as demonstrator of anatomy, desire your acceptance of a Seal Ring, and with it, their earnest wishes for your welfare.

Sincerely yours,

G. J. SWEET, C. C. DELLENBAUGH,
H. R. STAGG, J. P. SHUMWAY,
N. L. BATES, W. W. POTTER,
L. DAMAINVILLE, and many others.

*Dr. Lemon's Reply.*—Gentlemen: For your kind remembrance, and handsome token of consideration and esteem, accept my warmest thanks.

In the pursuit of the science of anatomy, we have many difficulties to encounter and many sacrifices to make, but, gentlemen, if we are ever to be true physicians, a knowledge of it is indispensable. Through that charnel house, the dissecting room, all have passed who have attained real excellence in our noble profession—a long list of honorable names, extending through thousands of years to the present time; a very large majority of you, I am happy to add, have appreciated the benefits of a practical application.

That another session may find you all in your old places, and with renewed vigor, exploring the still hidden regions of science, is the wish of your sincere friend.

BENJ. H. LEMON.

To H. R. Stagg, N. L. Bates, G. J. Sweet, C. C. Dellenbaugh, L. Damainville, W. W. Potter, J. P. Shumway, and many others.

